# Minimized Triple-Band Eight-Element Antenna Array for 5G Metal-frame Smartphone Applications

**DOI:** 10.3390/mi13010136

**Published:** 2022-01-15

**Authors:** Jianlin Huang, Zhuoni Chen, Qibo Cai, Tian Hong Loh, Gui Liu

**Affiliations:** 1 College of Electrical and Electronics Engineering, Wenzhou University, Wenzhou 325035, China; 194511981414@stu.wzu.edu.cn (J.H.); 20451941037@stu.wzu.edu.cn (Z.C.); caiqibo@wzu.edu.cn (Q.C.); 2 National Physical Laboratory, Teddington TW11 0LW, UK; tian.loh@npl.co.uk

**Keywords:** 5G new radio (5G NR), sub-6 GHz, MIMO, triple-band antenna, metal-frame smartphone

## Abstract

A multiple-input-multiple-output (MIMO) antenna array for triple-band 5G metal-frame smartphone applications is proposed in this paper. Each single antenna element consists of an S-shaped feeding strip and an L-shaped radiation strip on the metal frame. The dimension of the antenna element is only 6.5 mm × 7 mm (0.076 λ_0_ × 0.082 λ_0_, λ_0_ is the free-space wavelength at the frequency of 3.5 GHz). The −6 dB impedance bandwidth of the proposed eight-antenna array can cover 3.3–3.8 GHz, 4.8–5 GHz, and 5.15–5.925 GHz. The evolution design and the analysis of the optimal parameters for a single antenna element are derived to investigate the principle of the antenna. The measured total efficiency is larger than 70%. The measured isolation is better than 13 dB. The measurements of the prototype agree well with the simulation results.

## 1. Introduction

5G mobile communication technology has plenty of advantages, such as high communication capacity, high mass connection density, and high transmission rate. However, with the limited internal space of the universal smartphones, the number of antenna elements will be influenced. In order to solve this problem, each antenna element should be minimized [1,2,3,4], and operate at dual-band [5,6,7,8,9,10,11,12] or broadband [13,14,15,16,17]. By sharing a common grounding branch for the two adjacent antenna elements, a compact self-decoupled antenna pair was obtained in [1,2,3]. In [4], the height of the small printed circuit boards (PCBs) placed vertically to the system PCB is only 3.8 mm, which is a novelty for compact antennas. By using a multi-slot decoupling technique, a dual-band eight-element antenna array is proposed in [5,12]. A single antenna element consisting of two radiators was presented for dual-band operation [6]. A folded monopole and a gap-coupled loop branch were combined together to obtain two broad bands [7]. By adjusting the impedance ratio of the stepped impedance resonators and the position of the microstrip feed line, a dual-band eight-antenna array is proposed for the 5G mobile application [8]. Two double-branch monopoles and a T-shaped decoupling stub were utilized for dual-band operation [9]. In [10], two different antenna elements, namely the folded L-shaped antenna and the couple-fed U-shaped antenna, were applied for LTE band 42 and 5.8-GHz WLAN band, respectively. A modified E-shaped strip on the corner of the frame was employed to realize a dual-band MIMO antenna [11]. Wideband MIMO antenna array can cover 5G sub-6 GHz bands, which was a good option for smartphones [13,15,16,17]. A systematic design of a high-performance eight-element antenna array was introduced for broadband operation [14].

On the other hand, isolation between antenna elements should be optimized for better performance [18,19,20,21,22]. A self-isolated MIMO antenna system was introduced in [18], which was achieved by introducing two vertical stubs into the original antenna element. By using a novel isolation technique named building block, a gap-coupled loop antenna and a loop antenna are isolated from each other [19]. A novel balanced open-slot antenna is designed as an array antenna element generating a balanced slot mode that can enhance the isolation [20]. The parasitic elements and defective ground can be used to obtain an outstanding isolation [21]. Different from the above isolation techniques, the author utilized a pattern diversity that two adjacent ports worked simultaneously but were excited at a relative phase in [22], which was proved to have good isolation.

This paper proposes a MIMO antenna array for triple-band 5G metal-frame smartphone applications. An S-shaped feeding strip and an L-shaped radiation strip on the frame are utilized to achieve good performance. By using an S-shaped feeding strip, we can improve the bandwidth of the lower frequency band with minimized size. The structure of the antenna element is only 6.5 mm × 7 mm, which is the innovative point of this paper. The −6 dB impedance bandwidth of the proposed eight-antenna array can fully cover 3.3–3.8 GHz, 4.8–5 GHz, and 5.15–5.925 GHz frequency bands.

## 2. Antenna Geometry

**Figure 1:**
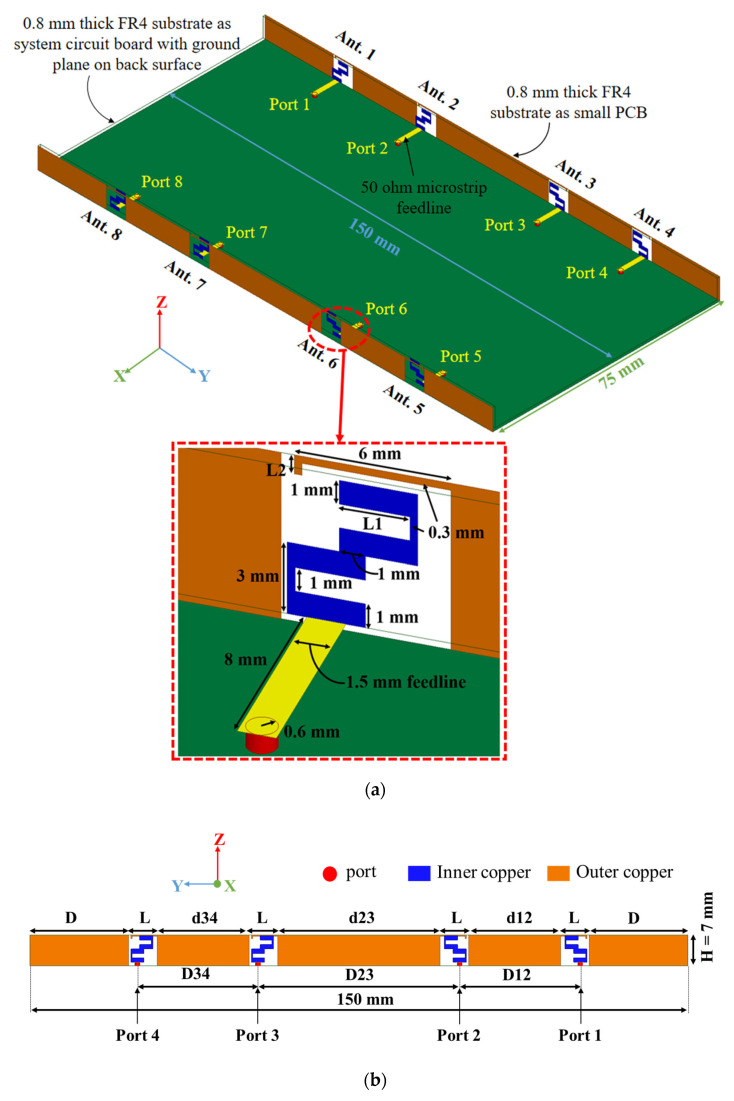
Geometry and dimensions of the proposed triple-band eight-element antenna array in millimeters: (**a**) Prospective view; (**b**) Side view.

The detailed geometry of the proposed triple-band eight-element array for 5G metal-frame smartphone applications is shown in Figure 1. There are two kinds of PCBs in the proposed MIMO antenna, including the mainboard and two sideboards. The dimensions of the mainboard and the sideboards are 150 mm × 75 mm × 0.8 mm and 150 mm × 6.2 mm × 0.8 mm, respectively. The system ground plane is printed on the bottom of the mainboard. In addition, there is a metal frame on the edge plate of each sideboard. The eight antenna elements are printed on the two sideboards which are placed vertically to the mainboard. The sideboards and mainboard are all printed on a FR4 substrate with ε_r_ = 4.4, and tanδ = 0.02, which are bonded to each other by tin. Each antenna is fed with a 50-Ω microstrip feedline and a SMA connector via a hole from the backside of the main substrate. The proposed antenna array has advantages of small volume (the single antenna element is only 6.5 mm × 7 mm) and triple-band operation (the −6 dB impedance bandwidth can fully cover 3.3–3.8 GHz, 4.8–5 GHz, and 5.15–5.925 GHz). Furthermore, a side view of the four antenna elements is given in Figure 1b. The values of the parameters are: L = 6.5 mm; H = 7 mm; D = 22.5 mm; d34 = d12 = 21 mm; d23 = 37 mm; D34 = D12 = 27.5 mm; and D23 = 46 mm.

## 3. Antenna Analysis

In order to understand the mechanism of the proposed MIMO antenna system, the design evolution, simulated surface current distribution, and the optimal parameter analysis of the antenna element have been studied.

**Figure 2:**
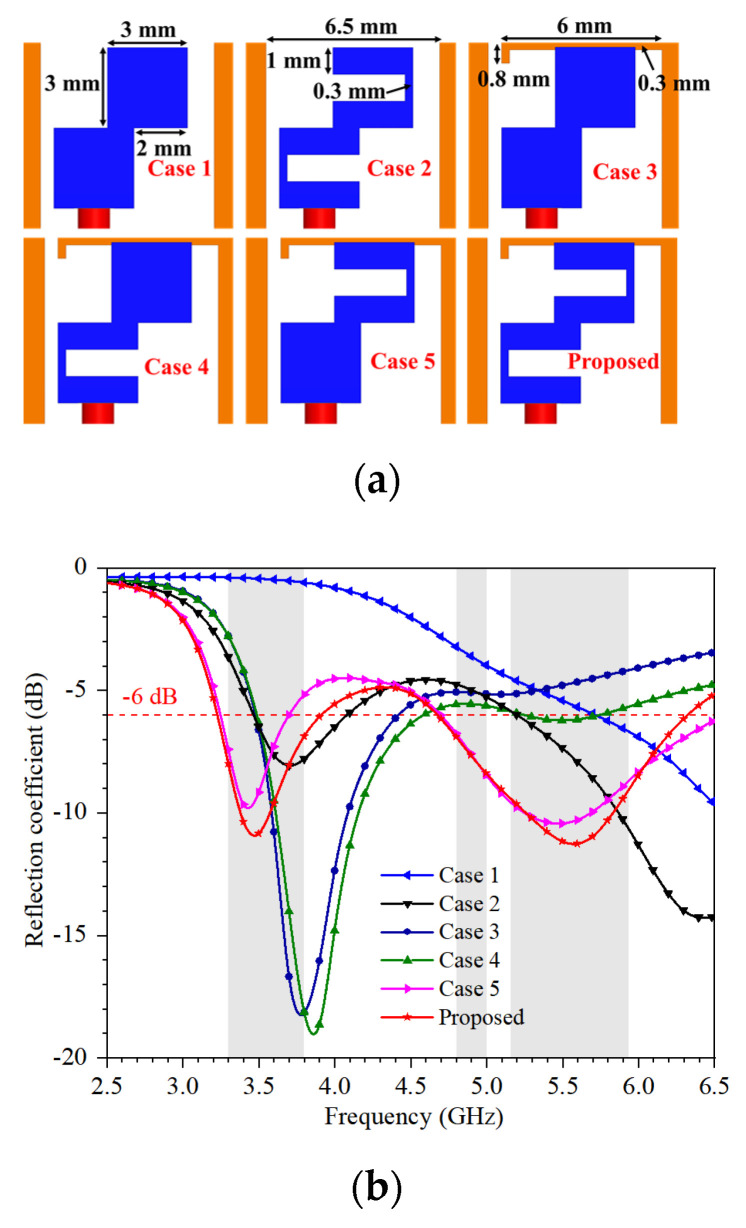
Design evolution of antenna element: (**a**) The evolution shapes; (**b**) The reflection coefficient.

In Figure 2, Case 1 is a simple monopole. By cutting two rectangle slots on the strips of antenna of Case 1, the antenna of Case 2 is obtained. By adding an L-shaped radiation strip on the frame of antenna of Case 1, the antenna of Case 3 is formed that has the character of single band at 3.8 GHz. By cutting a rectangle slot of the antenna of Case 3, the antenna of Case 4 is produced, which gets a better performance compared to that of Case 3. While cutting another rectangle slot of antenna of Case 3, the antenna of Case 5 is formed. By combining the superiority of antennas of Case 4 and Case 5, the proposed antenna element is proposed, which has two resonance points that can fully cover 3.3–3.8 GHz in the lower frequency band and 4.8–5.925 GHz in the higher frequency band.

**Figure 3:**
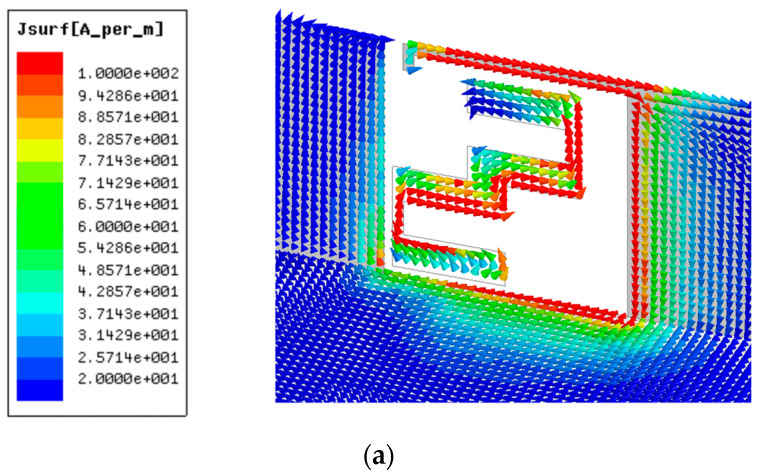

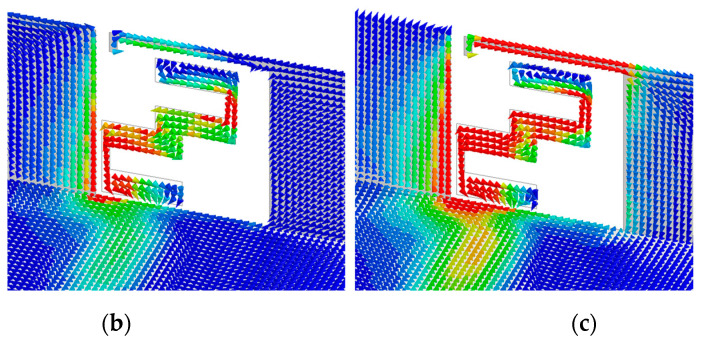
Simulated surface current distribution at (**a**) 3.5 GHz, (**b**) 4.9 GHz, and (**c**) 5.5 GHz.

Figure 3 show the surface current distribution simulated at 3.5 GHz, 4.9 GHz, and 5.5 GHz. The distribution of the surface current at 3.5 GHz is mainly concentrated at the right side of the frame. On the contrary, the surface current at 4.9 GHz is focused on the left side of the frame. However, the surface current distribution at 5.5 GHz is on the left and top sides.

**Figure 4:**
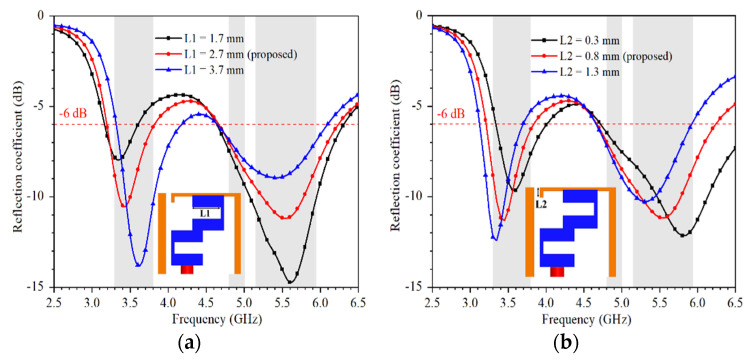
The simulated reflection coefficient as a function of (**a**) L1, (**b**) L2.

Figure 4 shows the simulated reflection coefficient as a function of L1 and L2. The value of L1 can be effectively used to change the resonant frequency of the lower frequency band, while the value of L2 can be utilized to tune the resonant frequencies of both the lower and higher frequency band.

## 4. Experimental Results and Discussion

**Figure 5:**
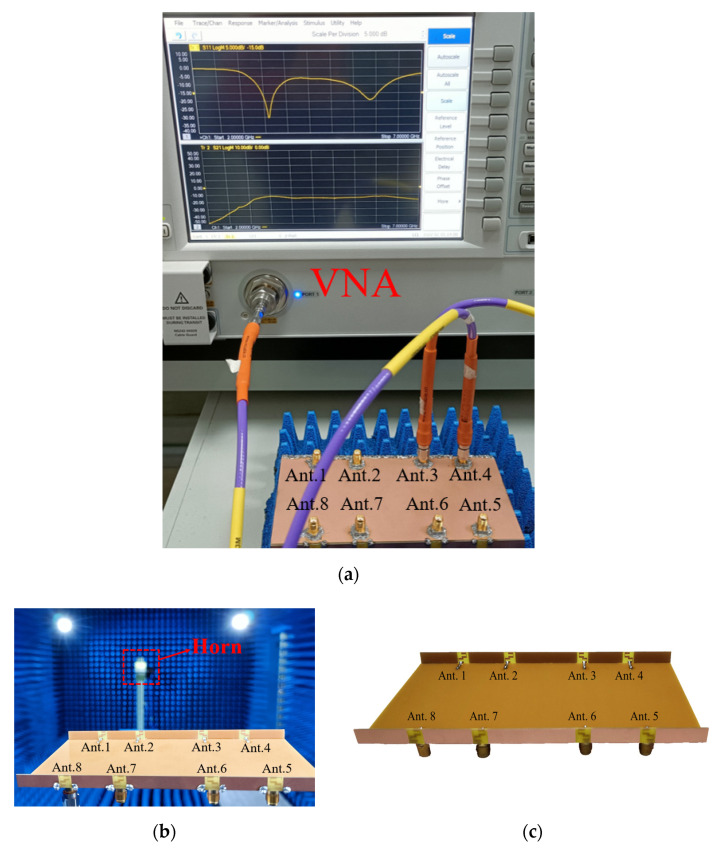
Photograph of the measurement setup; (**a**) vector network analyzer; (**b**) anechoic chamber; (**c**) fabricated antenna.

To verify the proposed design, an antenna prototype was fabricated using the optimized dimensions described in Figure 1. Figure 5 is a photograph of the measurement setup.

**Figure 6:**
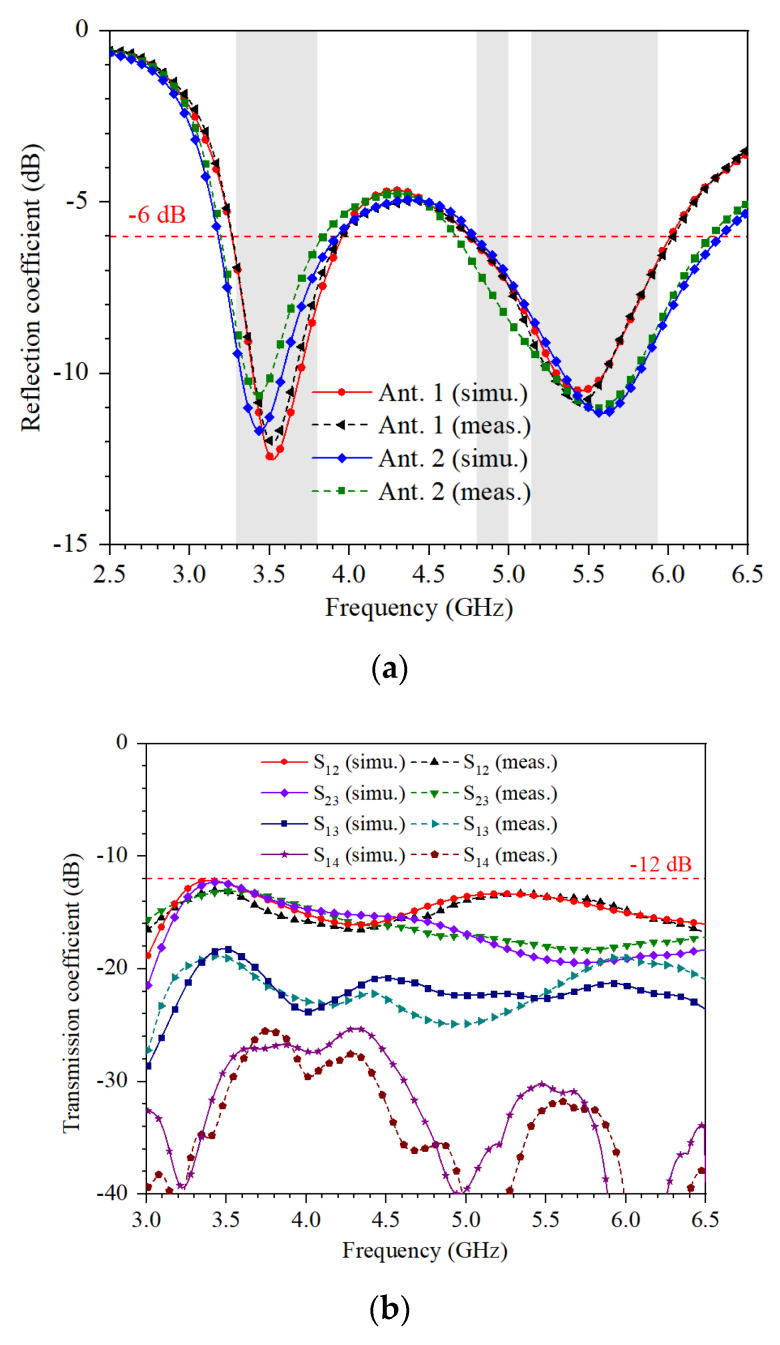
Simulated and measured coefficients of the proposed antenna array: (**a**) simulated and measured reflection coefficient; (**b**) simulated and measured transmission coefficient.

Figure 6 shows the simulated and measured coefficients. The antenna is measured by Keysight Vector Network Analyzer N5224A. It can be seen that the simulated and measured bandwidth can cover 3.3–3.8 GHz, 4.8–5 GHz, and 5.15–5.925 GHz. The isolation between different ports is larger than 13 dB. The slight frequency offset is due to excess solder.

**Figure 7:**
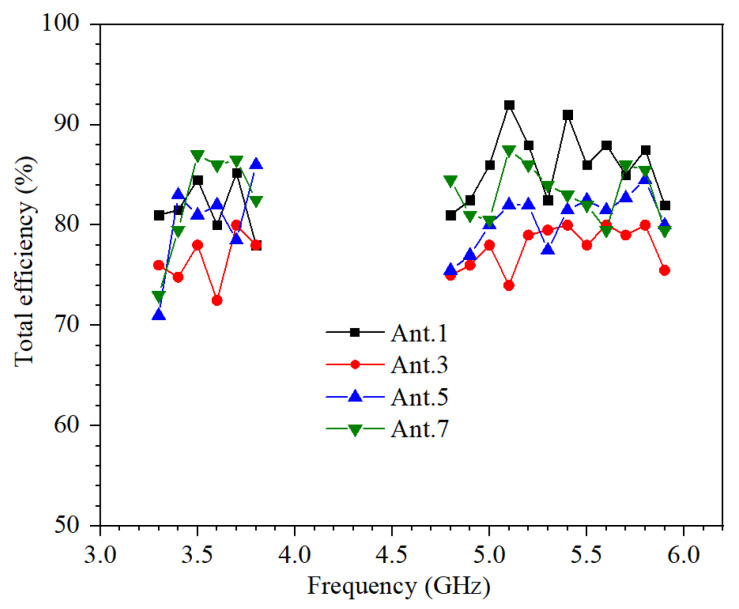
Measured total efficiencies of Ant. 1,2,3,4.

Figure 7 shows all measured total efficiencies which are larger than 70%. The inconsistent efficiency of symmetrical antennas is due to the current skin effect caused by excess solder.

In this study, the imaginary part and real part of the S-parameters are measured by VNA, and the calculated envelope correlation coefficient (ECC) of the proposed eight-antenna MIMO system according to Equation (1).

(1) $$\mathrm{ECC}=\frac{\iint_{4\pi }A_{\mathrm{ij}}(\theta ,\phi )\sin(\theta )d\theta d\phi }{\sqrt{\iint_{4\pi }A_{\mathrm{ii}}(\theta ,\phi )\sin(\theta )d\theta d\phi \iint_{4\pi }A_{\mathrm{ij}}(\theta ,\phi )\sin(\theta )d\theta d\phi }}$$

**Figure 8:**
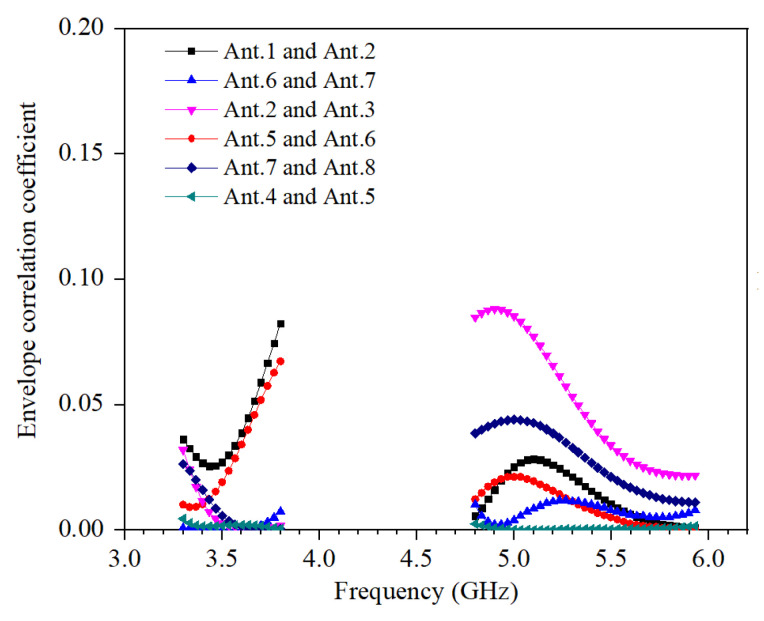
Calculated ECC.

The calculated ECC of the proposed MIMO antenna system is shown in Figure 8. The values of ECC in the operating frequency bands are smaller than 0.09.

The calculated diversity gain (DG) is related to ECC in Equation (2).

(2) $$\mathrm{DG}=10\;(\mathrm{dB})\;\times \sqrt{1-{\left|\mathrm{ECC}\right|}^{2}}$$

**Figure 9:**
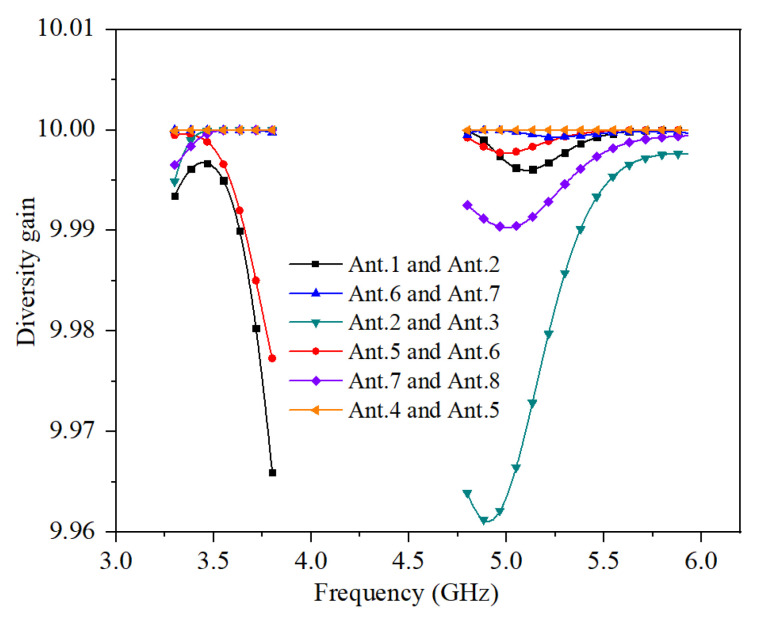
Calculated DG.

The calculated DG of the proposed MIMO antenna system is shown in Figure 9. The values of DG in the operating frequency bands are greater than 9.96.

The multiplexing efficiency (ME) is related to ECC in Equation (3). η_1_ and η_2_ are total efficiencies in Figure 7.

(3) $$\mathrm{ME}=\sqrt{\eta_{1}\eta_{2}(1-{\left|\mathrm{ECC}\right|}^{2})}$$

**Figure 10:**
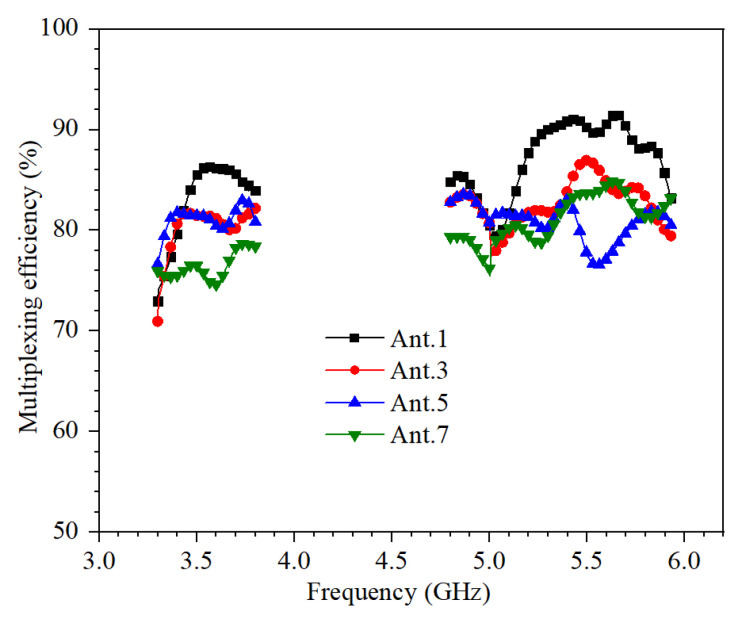
Calculated ME.

The calculated ME of the proposed MIMO antenna system is shown in Figure 10. The values of ME in the operating frequency bands are greater than 70%.

The channel capacity loss (CCL) is calculated from S-parameters using MATLAB in Equations (4) and (5).

(4) $$\mathrm{CCL}=-\log_{2}\left[\det(\Psi )\right],\;\Psi =\left[\begin{array}{cc} P_{11} & P_{12}\\ P_{21} & P_{22} \end{array}\right]$$

(5) $$P_{\mathrm{ii}}=1-{\left|S_{\mathrm{ii}}\right|}^{2}-{\left|S_{\mathrm{ij}}\right|}^{2},\;P_{\mathrm{ij}}=-{{(S}_{\mathrm{ii}}}^{*}S_{\mathrm{ij}}{{+S}_{\mathrm{ji}}}^{*}S_{\mathrm{jj}})\;;\;\mathrm{for}\;i,\;j=1\;\mathrm{or}\;2$$

**Figure 11:**
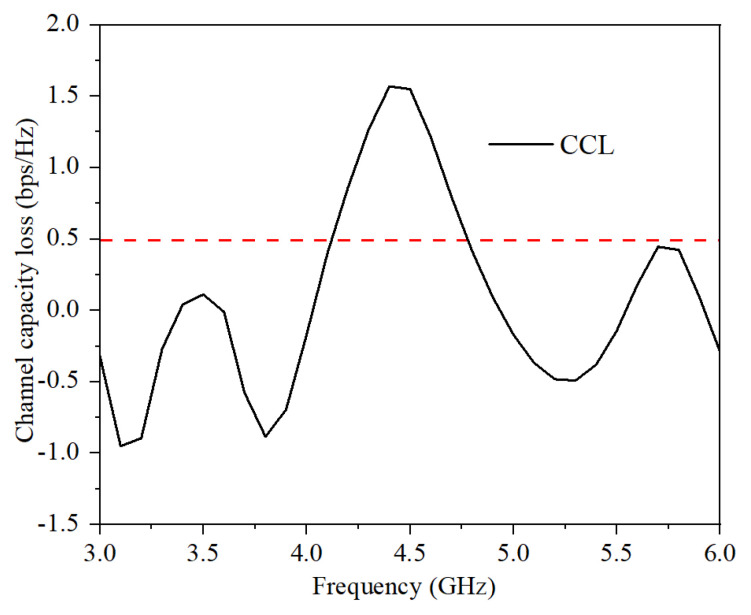
Calculated DG.

The calculated CCL of the proposed MIMO antenna system is shown in Figure 11. The values of CCL in the operating frequency bands are smaller than 0.5.

The total active reflection coefficient (TARC) is calculated from S-parameters in Equation (6).

(6) $$\mathrm{TARC}=\sqrt{\frac{{{(S}_{11}{{+S}_{21}}^{*})}^{2}{+(S}_{21}{{+S}_{22}}^{*}{)}^{2}}{2}}$$

**Figure 12:**
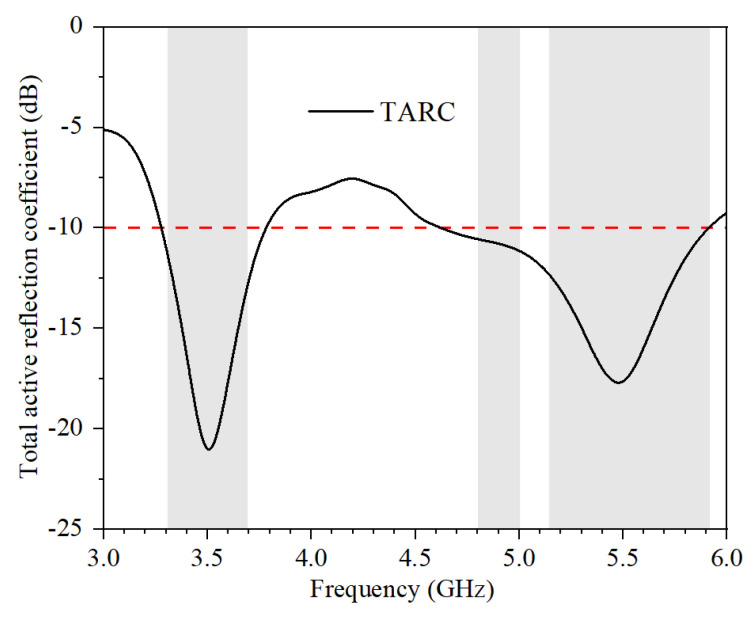
Calculated TARC.

The calculated TARC of the proposed MIMO antenna system is shown in Figure 12. The values of TARC in the operating frequency bands are smaller than −10 dB.

**Figure 13:**
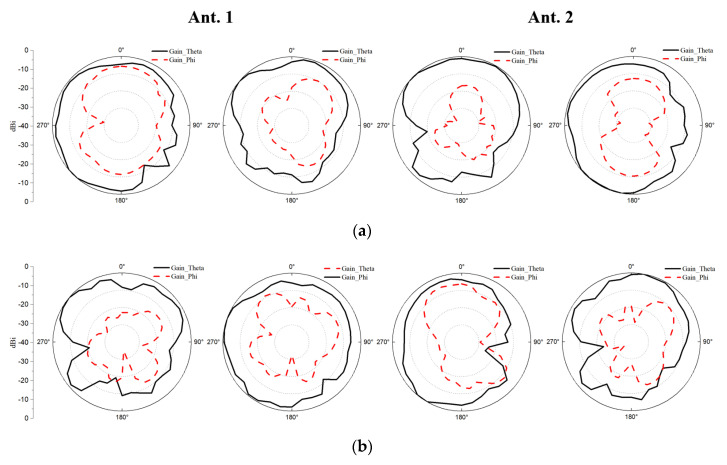

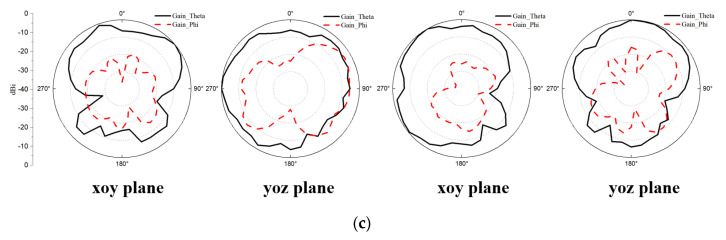
Measured 2D radiation patterns of Ant. 1 and Ant. 2 at (**a**) 3.5 GHz. (**b**) 4.9 GHz. (**c**) 5.5 GHz.

The radiation patterns of the proposed antenna element at 3.5 GHz, 4.9 GHz, and 5.5 GHz are shown in Figure 13. The measured co-pol and cross-pol are represented by solid and dashed lines, respectively. The cross-pol of all antennas is smaller than the co-pol. The co-pol and cross-pol of the same antenna are different at disparate frequency points. Since Ant.1 and Ant.2 have the same shape and different positions, radiation patterns are slightly similar, but not identical.

In Table 1, the proposed eight-element antenna array not only has the advantage of the comprehensive performance, but also has a minimized size. Therefore, it can be well employed in the future triple-band ultra-thin 5G mobile phones.

## 5. Conclusions

In this paper, a minimized triple-band eight-element antenna array covering 3.3–3.8 GHz, 4.8–5 GHz, and 5.15–5.925 GHz has been proposed. The single antenna element consists of an S-shaped feeding strip and an L-shaped radiation strip on the metal frame. The size of a single antenna element is only 6.5 mm × 7 mm, which is the innovation point of this paper. The measured total efficiencies are larger than 70%, and the isolations are better than 13 dB, respectively. The antenna was verified by both simulation and measurement. The proposed antenna element has the advantage of comprehensive performance under the minimum size. Therefore, it is a good candidate for 5G mobile handsets.

## Figures and Tables

**Table 1:** Comparison of the 5G MIMO smartphone antennas.

Reference	Operating Band (GHz)	Isolation(dB)	ECC	Efficiency (%)	Size(mm^3^)
[5]	3.4–3.8 4.8–5 (−6 dB)	>15.5	<0.06	40–85	15.2 × 7 × 0.8
[6]	3.3–3.6 4.8–5 (−10 dB)	>12	<0.15	>45	10.6 × 5.3 × 0.8
[7]	3.4–3.6 4.8–5.1 (−6 dB)	>11.5	<0.05	40–85	15 × 7 × 0.8
[8]	3.4–3.6 5.15–5.925 (−10 dB)	>11.2	<0.08	51–59	10 × 10 × 0.8
[9]	3.3–4.2 4.8–5 (−6 dB)	>10	<0.1	53.8–79.1	18.6 × 7 × 0.8
[10]	3.4–3.6 (−6 dB) 5.72–5.875 (−10 dB)	>17.1	<0.045	45–62	14.9 × 7 × 0.8
[11]	3.3–3.8 4.8–5 5.15–5.925 (−10 dB)	>18	<0.06	60–75	15.8 × 7 × 0.8
[13]	3.3–6 (−10 dB)	>10	<0.1	40–70	13.9 × 7 × 0.8
Pro.	3.3–3.8 4.8–5 5.15–5.925 (−6 dB)	>13	<0.1	>70	6.5 × 7 × 0.8

## Data Availability

The data presented in this study are available on request from the corresponding author.
